# Microspheres Based on Blends of Chitosan Derivatives with Carrageenan as Vitamin Carriers in Cosmeceuticals

**DOI:** 10.3390/polym16131815

**Published:** 2024-06-26

**Authors:** Kamila Lewicka, Anna Smola-Dmochowska, Piotr Dobrzyński, Natalia Śmigiel-Gac, Katarzyna Jelonek, Monika Musiał-Kulik, Piotr Rychter

**Affiliations:** 1 Faculty of Science and Technology, Jan Dlugosz University in Czestochowa, 13/15 Armii Krajowej Av., 42-200 Czestochowa, Poland; k.lewicka@ujd.edu.pl (K.L.); p.dobrzynski@ujd.edu.pl (P.D.); 2 Centre of Polymer and Carbon Materials, Polish Academy of Sciences, 41-819 Zabrze, Poland; asmola@cmpw-pan.pl (A.S.-D.); ngac@cmpw-pan.pl (N.Ś.-G.); kjelonek@cmpw-pan.pl (K.J.); mmusial@cmpw-pan.pl (M.M.-K.)

**Keywords:** chitosan Schiff base, κ-carrageenan, microspheres, controlled release, vitamin, cosmetic formulations, trans-epidermal absorption

## Abstract

Chitosan (CS) has a natural origin and is a biodegradable and biocompatible polymer with many skin-beneficial properties successfully used in the cosmetics and pharmaceutical industry. CS derivatives, especially those synthesized via a Schiff base reaction, are very important due to their unique antimicrobial activity. This study demonstrates research results on the use of hydrogel microspheres made of [chitosan-*graft*-poly(ε-caprolactone)]-*blend*-(ĸ-carrageenan)], [chitosan-2-pyridinecarboxaldehyde-*graft*-poly(ε-caprolactone)]-*blend*-(ĸ-carrageenan), and chitosan-sodium-4-formylbenzene-1,3-disulfonate-*graft*-poly(ε-caprolactone)]-*blend*-(ĸ-carrageenan) as innovative vitamin carriers for cosmetic formulation. A permeation study of retinol (vitamin A), L-ascorbic acid (vitamin C), and α-tocopherol (vitamin E) from the cream through a human skin model by the Franz Cell measurement system was presented. The quantitative analysis of the release of the vitamins added to the cream base, through the membrane, imitating human skin, showed a promising profile of its release/penetration, which is promising for the development of a cream with anti-aging properties. Additionally, the antibacterial activity of the polymers from which the microspheres are made allows for the elimination of preservatives and parabens as cosmetic formulation ingredients.

## 1. Introduction

The growing application versatility of biocompatible and biodegradable polymers makes them very attractive materials as an alternative for conventional, traditional polymers in many industrial branches, including packaging, agriculture, medicine, health care, and cosmetics [1,2,3,4,5,6,7]. Although chitosan (CS) use in cosmetics is a relatively new challenge, more and more scientists are becoming interested in this field. Its various unique features, such as biocompatibility, non-toxicity, and biological activity, including antimicrobial and antioxidant properties, make this polymer more and more attractive, especially in skin care applications [8,9].

Chitosan as a polycationic biopolymer derived through the alkaline deacetylation of chitin is an ingredient approved for use in cosmetics by both the FDA and the EU [10]. Chitosan has a film-forming function and, additionally, by creating a hydrophilic film on the skin, it prevents water loss and, therefore, can be used as a skin moisturizer, thickener, and emulsion stabilizer [8,9]. These properties contribute to the fact that chitosan is used in cosmetic masks, which work on a similar principle to dressings. Additionally, it can be a component of emulsions, gels, foams, and aerosols of cosmetics intended for use on skin, nails, and hair, or in oral hygiene preparations.

The antimicrobial effects of chitosan occur in two mechanisms of action. Directly, its addition to cosmetic preparations inhibits the development of microorganisms, e.g., in oral hygiene preparations, where bacteria play a key role in the development of dental plaque, or in deodorants and antiperspirants. CS decreases and sweat bacteria develop, leading to a reduction in metabolite formation responsible for the unpleasant smell. Indirectly, the addition of chitosan into cosmetic formulations reduces the use of preservatives [1,11,12,13,14,15].

Since there is a constant demand for new solutions and new ingredients in the cosmetics industry, there are a huge number of modifications of chitosan, mainly chemical ones.

Additionally, a promising direction for the use of chitosan in the cosmetics market is its use for the encapsulation of active ingredients. Chitosan in this form not only extends the durability of the cosmetic product by protecting the active ingredients against degradation but also provides time-controlled release when treated on the skin [10,16].

Another natural raw material used in the cosmetics industry is carrageenan (CG). Carrageenan is a biocompatible, biodegradable sulfated plant polysaccharide with diverse biological activities, like antioxidant, anti-aging, anti-photoaging, and anti-melanogenesis features. In addition, CG can increase the viscosity of solutions, play the role of gelling agents and emulsion stabilizers, and is characterized by high water-holding capacity and mechanical strength of the gel; therefore, it is used in toothpaste, creams, ointments, soaps, shampoos, body lotions, gels, and foams [17,18,19]. Carrageenan interacts with human carotene to give soft skin in the body, hand lotions, and silky hair in shampoos. Is non-toxic and can be used as an alternative to lightweight oils, such as acetyl alcohol, or silicone-derived ingredients [20].

Blends of both polysaccharides were already successfully used as hydrogel carriers of bioactive substances. Grenha et al. (2010) developed chitosan- and carrageenan-based nanoparticles for the release of a model protein, ovalbumin. The authors obtained regular shaped and smooth surfaced, non-cytotoxic (in the concentration range from 0.1 to 3 mg/mL) nanocarriers able to control the release of protein for up to 3 weeks [21]. Naiel et al. (2023) developed oil-trapped polymer beads based on alginate (Alg)/iota-carrageenan (i-CG) covered with a layer of aminated chitosan (AmCs) for the sustained release of amoxicillin trihydrate (AMT). In vitro biological evaluation showed that the obtained structures demonstrated antimicrobial activity and significant biodegradability under physiological enzymatic conditions, as well as prolonged antibiotic release for up to 25 h without the burst effect. Furthermore, cytotoxicity assessment confirmed the safety of the formulated polymer beads, suggesting their potential use as effective carriers for the oral administration of antibiotics [22]. Stavarache et al. (2021) investigated particle production by cross-linking chitosan (CS) with sodium tripolyphosphate (TPP) and the effect of the k-carrageenan (kCG) layer. The biocompatibility test of the developed materials showed good viability of CCD 841 CoN cells cultured in contact with all the beads contained in the particles suspended in the kCG solution. Moreover, cells cultured in contact with all hydrogel beads showed an elongated phenotype, which indicated low cytotoxicity and no negative impact on cell behavior [23]. Similarly, the study by Athipornchai et al. (2024) presented a hydrogel system with a controlled release of the bioactive compound—mangiferin. Mangiferin-loaded core particles were prepared from a κ-carrageenan solution, which was then ionically cross-linked and encapsulated in chitosan (efficiency of 85%). The biocompatibility test performed on all hydrogel samples showed good viability of L929 and Vero cells with low cytotoxicity [24].

Modern dermo-cosmetics, based on polymeric nano- and microparticles or nanofibers containing biologically active fibers, play a new role and are gradually becoming a normal market product. Nanoparticles or microparticles allow for the improvement of the physicochemical properties of classic cream emulsions by increasing the penetration of bioactive compounds into the deep layers of the skin. It becomes possible to maintain optimal skin hydration and elasticity, balance of the cell matrix, and other functions related to the appearance of the skin because nanocarriers can offer an “arsenal” of different loaded particles. Polymeric nanoparticles are a proven non-invasive way of administering factors that not only care for the skin but also various other stimulants and systemic drugs through, e.g., hair follicles [25]. Recently, there has been a growing interest in the use of chitosan and carrageenan as a carrier medium for various vitamins. Liping et al. (2020) prepared a chitosan/vitamin C complex as a multifunctional raw material for cosmetics [26]. Zhang et al. (2022) prepared a thermo-sensitive chitosan–vitamin C hydrogel scaffold to reduce oxidative stress injury after myocardial infarction [27]. Jiao et al. (2018) received chitosan-coated liposomes with the encapsulation of vitamin C and folic acid as a delivery system for the antioxidant system [28]. Yee et al. (2016) obtained carrageenan hydrogels as a sustained release matrix for the delivery of tocotrienol-rich palm-based vitamin E [29]. Kasapis et al. (2015) developed a carrier of ĸ-carrageenan/glucose syrup to control the release of thiamin (Vitamin B1) [30]. Buchholz et al. (2015) reported modified-release matrix tablets based on carrageenan combined with microcrystalline cellulose and lactose, containing vitamin B2 [31]. Human skin is exposed to oxidative damage caused by free radicals, which is one of the main causes of skin aging. Many bioactive antioxidants, such as vitamins A, E, and C, are not able to be produced endogenously and so must be provided externally or through diet. L-ascorbic acid is the most abundant antioxidant in the skin, fighting free radicals, and is a biologically active form of vitamin C [32].

Vitamin C (VC) is an antioxidant responsible for effectively neutralizing the formation of hazardous free radicals in the human body [11,33]. It is also used as an anti-aging ingredient due to its potential to stimulate collagen biosynthesis and stability by hydroxylation reactions, improving the firmness and density of skin tissues [34]. Vitamin A (VA), most often used in cosmetics in the form of retinol or its derivatives, also has a strong antioxidant and anti-aging effect. VA prevents collagen degradation in the skin and leads to a clinical improvement in wrinkles [35]. Additionally, VA can quench singlet oxygen, a highly reactive free radical, which is formed in smog as a result of exhaust gases and UV rays. It has been found that VA and its derivatives in cosmetic products can normalize keratinization and regulate epithelial cell growth and differentiation, acne, and other specific skin disorders (psoriasis, ichthyosis, etc.) [34,36]. Retinol is currently the most popular VA ingredient in cosmetics [34]. Vitamin E is used in many photoaging skin care cosmetics due to its high antioxidant and protective activity and ability to improve the skin’s protective barrier [37]. VE prevents the oxidation of lipids, proteins, and DNA in the body, thus quenching the effects of free radicals [38]. Vitamin E relieves swelling caused by UV radiation and reduces erythema. In cosmetics, VE is used in the form of the most absorbable tocopherol [34].

A separate problem when formulating the compositions of cosmetic preparations, including various types of creams, is the need to provide them with protection against bacterial growth and biological contamination to provide long shelf life and microbiological safety. Contamination can occur through raw materials, poor manufacturing conditions, inadequate packaging, or improper storage. One of the most important antibacterial protections is the introduction of synthetic or natural preservatives into the composition of cosmetics. They must meet several criteria [39]. They should mostly demonstrate very good antimicrobial efficacy, no toxicity, and compatibility with the other ingredients in the cosmetic formulation [40]. The most valuable in the recipes of modern cosmetic preparations are substances with multifunctional properties, which, in addition to antibacterial activity, also play the role of chelating agents, surfactants, humectants, or antioxidants [41,42,43]. Unfortunately, many antibacterial effective substances used in cosmetics also have toxic or allergenic effects, which strongly limits their use [44,45]. For this reason, in the case of the production of creams or other skin care antibacterial products, to obtain the expected effectiveness of such formulations, in addition to natural products (oils, bee products), it is necessary to use much stronger substances, such as alkylamines and alkanolamines, aliphatic chlorides, and selected metals and their salts, as well as antibiotics [46]. The use of these substances in antibacterial formulations is common; however, they are quite controversial due to the high probability of adverse health effects and, in the case of the use of antibiotics, they can increase the probability of creating mutations of drug-resistant microorganisms.

This study presents the results of the formulation of polymer microspheres containing vitamins A, E, and C. They were formulated using compatible hydrogel blends composed of carrageenan and chitosan derivatives. Obtained blends are biodegradable hydrogels with a high water absorption capacity and demonstrate good antibacterial properties, which were described in detail in our previous publication [47]. The microspheres, being carriers of vitamins, play a simultaneous role as bacteriostatic agents, and for this reason, are promising ingredients of the typical moisturizing cream. The kinetics of the vitamin release and penetration through the skin were studied in vitro. The cytotoxicity of microspheres towards human skin cells, fibroblasts and keratinocytes, was also determined.

## 2. Materials and Methods

### 2.1. Reagents and Solutions

Low molecular weight chitosan (CS) with a degree of deacetylation (DDA) of 85% and ĸ-carrageenan (CG) with an average weight mass of about 500,000 g/mol were purchased from Merck Life Science (Darmstadt, Germany). Before the modification reactions, chitosan was subjected to additional deacetylation, obtaining DDA = 96%. Glutaraldehyde (50% (*v*/*v*) (GA); paraffin oil; Span 80; retinol (VA); L-ascorbic acid (VC); and α-tocopherol (VE) were purchased from Merck Life Science (Darmstadt, Germany) and used as received. Organic solvents were purchased and used as received (Chempur, Piekary Slaskie, Poland). Strat-MTM synthetic membranes were acquired from Merck Life Science (Darmstadt, Germany).

Polymer blends obtained from CS and CG and CS derivatives from CG were used for the microsphere’s preparation. Their description and abbreviations are presented in Table 1.

A detailed synthesis procedure of chitosan derivatives, their properties, including antibacterial activity, and the method of forming blends were described in a previous study [47].

### 2.2. Composition of the Cream Base

The release profile and transdermal diffusion of vitamins were studied from the moisturizing cosmetic cream enriched with an admixture of the obtained microspheres filled with vitamins. The cream recipe and its production procedure are presented below.

Ingredients (% by weight).

Phase A:

- Deionized water—61.8%, improves the texture and moisturizes the skin;
- Sodium Phytate (Merck, Rahway, NJ, USA)—0.2%, chelating agent (sequestrant);
- Propanediol (Merck, Rahway, NJ, USA)—5%, solvent, improves hydration, and regulates viscosity;
- Panthenol—1% (Merck, Rahway, NJ, USA), provitamin B5, humectant, and a soothing and regenerating agent;
- Xanthan Gum—1% (Merck, Rahway, NJ, USA); emulsifying, emulsion stabilizing, and a skin conditioning agent.

Phase B:

- Caprylic/Capric Acid Triglyceride (Cisme, Milan, Italy)—3%, emollient, and a viscosity regulator;
- Butyrospermum Parkii Butter (Clariant, Muttenz, Switzerland)—2%, emollient, moisturizing, and a conditioning agent;
- Prunus Amygdalus Dulcis Oil (Clariant, Muttenz, Switzerland)—5%, emollient, and a conditioning agent;
- Cetearyl Alcohol (Alfa Chemistry, Holbrook, NY, USA)—4%, emmolient and humectant;
- Polyglyceryl-6 Stearate Polyglyceryl-6 Behenate (Evonik, Piscataway, NJ, USA)—6%, emulsifier, and moisturizing;
- Pantolactone (Sigma-Aldrich, Saint Louis, MO, USA)—1%, humectant, and a moisturizing agent.

Phase C:

- Microspheres filled with vitamins (A, E, or C) suspended in butylene glycol—10%, a carrier containing biologically active vitamins, and a bacteriostatic and antifungal agent.

The components of phases A and B were placed in separate beakers and mixed thoroughly using a hand blender. The phase A component was mixed at room temperature, and phase B was in a beaker thermostated in a water bath at approximately 70–80 °C until uniform mixtures were obtained. Then, after heating both vessels to approximately 70 °C, phase A was poured into a small reactor placed on a water bath at a temperature of 80 °C, equipped with a high-speed mixer. Next, oil phase B was gradually dosed for approximately 2–3 min with constant intensive stirring. After cooling to room temperature, stirring velocity was significantly reduced, and phase C was added. A fluffy cream was obtained and stored in a refrigerator at 7–10 °C.

### 2.3. Forming the Microparticles

CS and CG blend microparticles were prepared by the w/o emulsion method followed by cross-linking using glutaraldehyde (GA). This method uses the reactive amine functional group of chitosan to cross-link with the aldehyde groups of the cross-linking agent. The proper amount of polymer blends was dissolved in 5 mL of a 2% acetic acid solution and dispersed in 50 mL of liquid paraffin with the addition of a few drops of Span 80 as a surfactant, with intensive mixing for 10 min using a mechanical stirrer. GA was added to the water–oil suspension in portions of 0.25 mL every 1 h for 4 h, and stirring was continued. At the end of 5 h, stirring was stopped and the microspheres were separated by vacuum-induced filtration, washed several times with n-hexane and diethyl ether by centrifugation at 6000 rpm, and then dried in a vacuum dryer at 40 °C.

Vitamin C-loaded microspheres were prepared by directly adding an aqueous solution of vitamin C to an acetic acid solution. In the case of vitamins A and E, their ethanolic solution was added to a 2% acetic acid. The initial vitamin content was 10% of the weight of the polymer blends.

### 2.4. Loading Efficiency of Vitamins

The loading efficiency (LE) was calculated according to the following equation:

(1) $$LE\left(\%\right)=\frac{Calculated\;vitamin\;concentration}{Theoretical\;vitamin\;concentration}\cdot 100\%$$

### 2.5. Morphology and Size Distribution of Microspheres

The shape and surface evaluation of microspheres was conducted by a Tescan model VEGA 3SBU scanning electron microscope (SEM) (Tescan Orsay Holding, Brno, Czech Republic). Surface measurement was conducted using accelerating voltage ranging between 1 and 3 kV. A scan analysis of the surface was conducted at low vacuum, and microspheres were not coated with a conductive layer. The distribution of microsphere dimensions was statistically calculated.

### 2.6. Study of Swelling and Solubility Degree of Microspheres

To investigate the swelling process and solubility of the obtained microspheres, the specified mass of the microspheres (W_d_) was packed in membrane sacks, and then each sample was placed in a vial filled with 10 mL of a pH 5.0 buffer at 34 °C on an incubated shaker. After the specified period, 3, 6, 9, and 12 h samples were withdrawn from the medium, and the mass of samples was weighted. The microspheres were dried by freeze-drying to a constant mass of W_d2_. The difference (W_d_ − W_d2_) was the amount of dissolved microsphere mass (M_s_). The relative mass loss (M_s_%) related to the solubility and degradation of the microspheres was calculated according to the following equation:

(2) $$M_{s}\%=\frac{{(W}_{d}-W_{d2})}{W_{d}}$$

The swelling ratio (SR) of the prepared microspheres in buffered water solution (pH 5.0) at a temperature of 34 °C after 12 h of incubation was calculated according to the following equation:

(3) $$SR=\frac{{(W}_{w}-W_{d})}{W_{d}}\cdot 100\%$$

where W_w_ is the weight of the wet microspheres sample; W_d_ is the weight of the dry samples before the experiments; and W_d2_ is the weight of the dry samples after incubation.

Then, the samples were freeze-dried until a constant mass was obtained.

### 2.7. In Vitro Vitamin Release and Trans-Epidermal Absorption Studies

The studies were carried out in a Franz Cell system with a diffusion area of 1.767 cm^2^ and a capacity of 7 mL for the receptor medium buffered to pH 5 (average physiologic skin pH value) [48]. The Franz Cell system was maintained at a constant temperature of 34 ± 0.5 °C (average facial skin temperature) [49], while the receptor medium was stirred constantly at 350 rpm. For the permeation studies, Strat-MTM synthetic membranes were used, and the donor part contained a typical basic moisturizing cream with the obtained microsphere carrier vitamin. Strat-MTM consists of two layers of polyethersulfone on top of one layer of polyolefin. These polymer layers create a porous structure with a gradient across the membrane in terms of pore size and diffusivity. The porous structure was impregnated with a proprietary blend of synthetic lipids, giving the synthetic membrane additional properties that imitate human skin. Aliquots of samples were collected at 15 min, 30 min, and 45 min and 1, 2, 3, 4, and 6 h. The conditions were maintained with the replacement of the same volume of receptor medium. All collected samples were analyzed by high-performance liquid chromatography (HPLC) using a Smartline System equipped with a pump, degasser, and UV detector (Knauer, Berlin, Germany). For the chromatographic separation of vitamins, an RP-C18 column packed with 5 μm (250 mm × 4.6 mm) shell particles was used at a column oven temperature of 25 °C. The mobile phase consisted of methanol/water (92:8) at a flow rate of 1.0 mL/min (isocratic elution for 20 min at 325 nm for VA, 254 nm for VC, and 292 nm for VE). The injection volume was 20 µL. To determine the actual concentration value, previously created calibration curves were used (Figure S1). Additionally, few representative elugrames are demonstrated in Figures S2 and S3.

### 2.8. Assessment of Cytotoxicity

Cytotoxicity testing was performed following the ISO 10993–5 standard. Human WI–38 fibroblasts (CCL–75), obtained from the American Type Culture Collection (ATCC), were cultured in Dulbecco’s Modification of Eagle’s Medium (DMEM) supplemented with 10% bovine serum (FBS), 100 U/mL penicillin, and 100 μg/mL streptomycin. Human keratinocytes (HaCaTs) were purchased from the Cell Line Service (CLS) and cultured in DMEM supplemented with 10% bovine serum (FBS), 100 U/mL penicillin, 100 μg/mL streptomycin, and 2 mM L-glutamine. A quantity of 10 mM [acid 4-(2-hydroxyethyl)-1-piperazineethanesulfonic acid] (HEPES) (pH 7.3) was also added to the experimental cultures. The cells were incubated at 37 °C and 5% CO_2_. Before cell culture, the materials were sterilized with a UV lamp. Each sample was placed in a vial, and DMEM was added to obtain a concentration of 1000 μg/mL. The samples were incubated at 37 °C for 24 h. After this time, dilution of the extract was obtained in the concentration range of 0.16–1000 μg/mL. To test for cytotoxicity, 100 μL of the cell suspension, containing 4 × 10^3^ cells, was transferred to the wells of 96-well plates and cultured in a standard medium for 24 h to ensure cell adhesion. After 24 h, the medium was replaced with a medium containing the extract of the tested material. Cells were incubated with the tested extracts for 72 h. Untreated cells were used as a negative control (K−), and cells treated with 5% DMSO were used as a positive control (K+). Cell viability was assessed using the Cell Counting Kit-8. Absorbance was read at 450 nm (reference: 650 nm). Statistical analysis was performed using the Statistica 10.0 program using one-way ANOVA. The results at the significance level of *p* < 0.05 were considered statistically significant.

## 3. Results

### 3.1. Microsphere Properties

The use of selected vitamins A, E, and C as the most popular agents in creams, ointments, and gels has certain limitations, related primarily to their solubility and photostability. In the case of vitamin C, the main challenge is maintaining its stability. It decomposes quickly in an aqueous environment, depending on the pH (faster at high values), and in the presence of metal ions and oxygen. Vitamin A and E are lipophilic vitamins and are chemically unstable and have poor ability to penetrate the skin, which limits their action in conventional preparations. New drug/cosmetics delivery system strategies currently in use aim to improve the optimization of the therapeutic agent by modifying their physicochemical properties or modulating their biopharmaceutical properties. Controlled release carriers like liposomes, solid lipid nanoparticles, microemulsions, microcapsules, and nanocapsules allow for the suppression of photodegradation and moisture action degradation of active, immobilized vitamins. They facilitate stability and improve skin permeability, increasing permeation into deep layers of the skin via prolonged release of the active substance [50].

For this reason, this study presents microsphere formulations based on polymer blends of chitosan and carrageenan as systems for the controlled release of selected vitamins. In our previous studies, we successfully synthesized biocompatible polymers with embedded Schiff bases as active antimicrobial agents. The introduction of PCL improved solubility in organic solvents, which is required in cosmetic preparations. The use of more hydrophobic carriers for active ingredients in cosmetics facilitates their stability and increases their availability to the skin. Blends with non-toxic carrageenan improved the swelling properties of chitosan-based polymers and showed promising antimicrobial activity, higher than plain chitosan [47].

Since the obtained polymer blends are very promising materials for potential use as controlled-release preparations of both hydrophilic drugs and hydrophobic cosmetic products, further research was carried out to formulate polymer carriers to study the degradation by the determination of the release kinetics of the selected vitamins through the membrane, imitating human skin.

**Figure 1:**
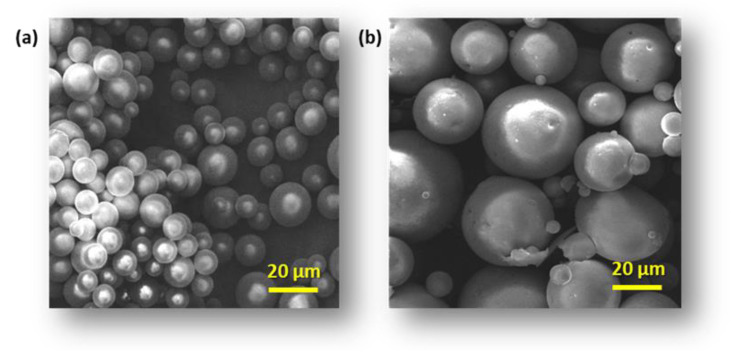
Scanning electron microscopy (SEM) images of microspheres CS-g-PCL(MSA):CG 50:50 loaded with VA vitamins (**a**) after receiving and (**b**) swelling microspheres after 12 h of incubation in apH 5.0 buffer.

**Figure 2:**
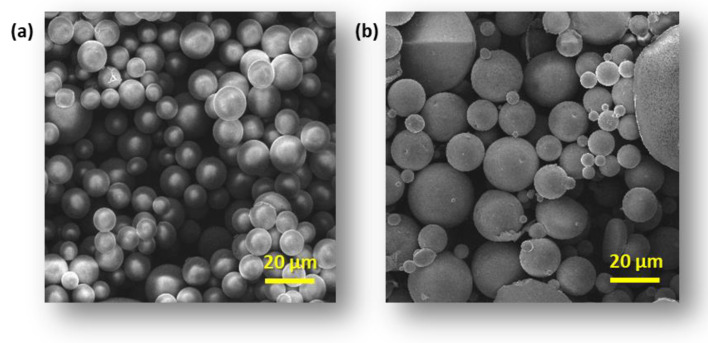
Scanning electron microscopy (SEM) images of microspheres CS-SB-PCA-g-PCL:CG 50:50 loaded with VA vitamins (**a**) after receiving and (**b**) swelling microspheres after 12 h of incubation in apH 5.0 buffer.

**Figure 3:**
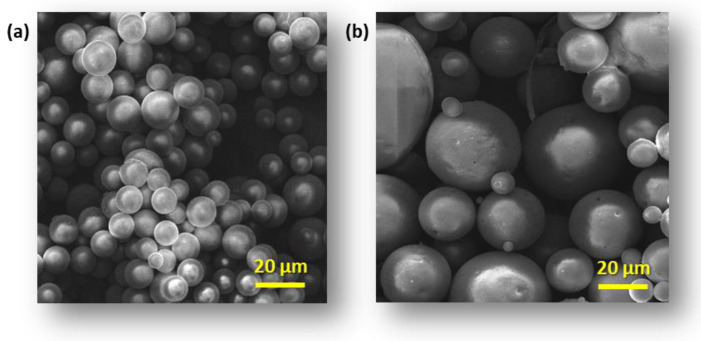
Scanning electron microscopy (SEM) images of microspheres CS-SB-SFD-g-PCL:CG 50:50 loaded with VA vitamins (**a**) after receiving and (**b**) swelling microspheres after 12 h of incubation in a pH 5.0 buffer.

**Figure 4:**
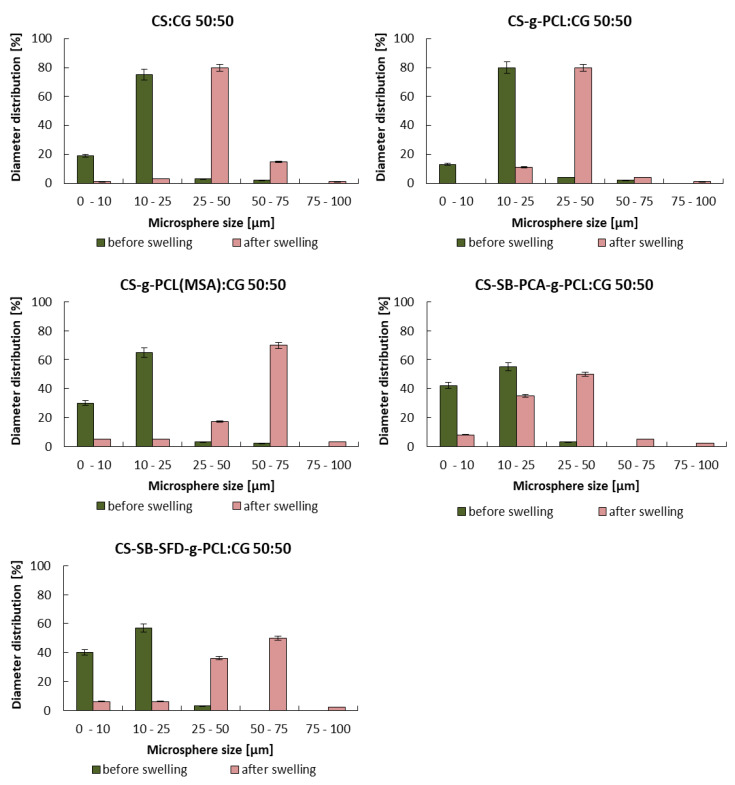
The diameter distribution of the microspheres loaded with vitamins after receiving (before swelling) and after 12 h of incubation in a pH 5.0 buffer.

The morphology of modified CS and CG blend microparticles was examined by SEM. Obtained microparticles have a regular spherical shape and a smooth surface (Figure 1a, Figure 2a and Figure 3a). The observation showed neither a lack of pores on the surface of the microspheres nor evidence of collapsed particles (Figure 4). Particle size is a key property in processes related to surface area and directly affects the degradation time of the polymer carrier and, therefore, the release process of the active substance. The formulation of particles with this size was possible by adjusting the mixing speed. The dimension of the obtained spheres also plays a role in transport through the pores in the skin. According to Flament et al. (2015) [51], the average pore size depends on age and genetics and is from 0.05 to 0.2 mm^2^; therefore, for effective transdermal transport, the diameter of the microspheres must be less than 120 µm. All types of microspheres, with or without vitamins, present a similar size, in the range of 10−20 μm in diameter (Figure 1, Figure 2 and Figure 3). No significant influence of the type of loaded vitamin on the shape and diameter distribution of the formed microspheres was observed. These parameters depended on the type of blend used to form them. SEM images obtained from particles after 12 h of incubation in a pH 5.0 buffer show that the microspheres formed with chitosan derivatives begin to swell and increase more than twice in diameter (Figure 1b, Figure 2b and Figure 3b). This process, of course, influenced the change in the dispersion characteristics of their diameters before and after swelling, which is presented in Figure 4. The dominant fractions of microspheres in this case have diameters ranging from 50 to 70 μm. This is a beneficial phenomenon from the point of view of the release of active ingredients loaded in matrices.

The percentages of loading efficiency (%LE) of the vitamin’s microspheres prepared are shown in Table 2. The %LE was obtained in the range of 70% to 97%. While there are no significant differences in the loading efficiency of individual polymer blends, there are noticeable differences in the loaded vitamins. LE was as follows: VA 93–97%, VC 70–76%, and VE 91–97%. This phenomenon is probably related to the similarity of the physical properties of hydrogel blends and behavior in the process of forming microspheres, as well as large differences in the water solubility between vitamins C and A or E.

### 3.2. Studies on the Degree of Swelling and Dissolution of Microspheres

**Figure 5:**
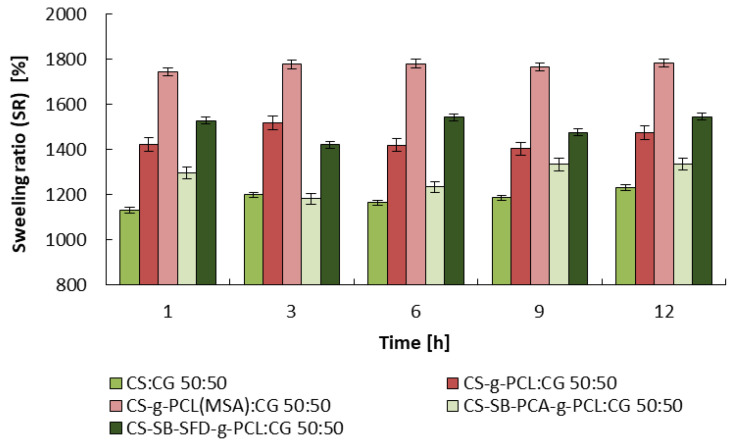
Swelling ratio (SR) of microspheres at different soaking times in a pH 5.0 buffer at 34 °C. Error bars represent the standard deviation of three replicates.

Microspheres obtained from the blends, similar to the material from which they were formed, are hydrogels that swell strongly in an aqueous environment. At the temperature of the facial skin and a pH value of approximately 5, their swelling degree (SR) depends on the soaking time and the type of material from which they were formed and ranges from 1200% to 1700%. The CS:CG 50:50 blend demonstrates the lowest swelling, and the highest was noted by CS-g-PCL(MSA):CG 50:50. The results of the swelling studies are shown in Figure 5.

**Figure 6:**
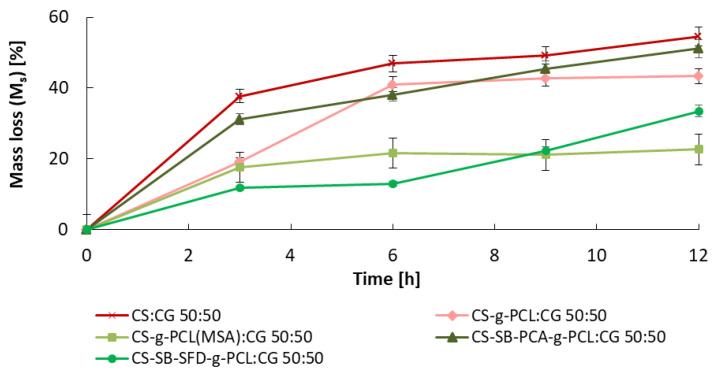
The relative mass loss (M_s_%) of microspheres after the incubation process in a pH 5.0 buffer at 34 °C. Error bars represent the standard deviation of three replicates.

The relative weight loss (M_s_%) of the different microsphere samples observed during incubation in the buffer solution is shown in Figure 6. Weight loss of the matrices is relatively slow throughout the degradation period. After 12 h of incubation in a pH 5.0 buffer, the matrices lost from 20% to 55% of their initial weight. The greatest weight loss of all CS:CG microspheres was observed for the blend composed of unmodified CS and CG, which is a result of the dissolution of pure polymers in the medium. The dissolution of polymer blends based on PCL-grafted chitosan samples, including Schiff bases, with carrageenan, was slower than pure CS and CG composition. The CS-g-PCL(MSA):CG 50:50 blends have the lowest weight loss ca 20%. This fact is related to the content of caproyl in the CS-g-PCL(MSA) copolymer and the much higher average length of the caproyl blocks compared to the analogous CS-g-PCL:CG 50:50 blends obtained in the ROP reaction initiated by the zinc(II) complex [40]. In the case of this composition, a rapid degree of degradation is noticeable for up to 6 h, with the degradation almost stopping later. Polymer blends containing a chitosan Schiff base show a good disintegration tendency. The degradation rate of the CS-SB-SFD-g-PCL:CG 50:50 blend reflected constant weight loss (up to 15%, after 6 h), and after that time, much faster degradation, and an almost doubled degradation measured by weight loss occurred. Similar behavior was noticed in the case of the second blend, CS-SB-PCA-g-PCL:CG 50:50, where the sample weight loss was almost 50% after 12 h.

The observed mass loss is mainly caused by the gradual degradation of polymer blends from which the microspheres were composed, generating the formation of fractions with increased solubility in water, which are able to leach out to the medium. The slowest weight loss (about 20–30% of the original weight) was noticed for microspheres obtained from blends with the lowest solubility in water, containing long caproyl chains (CS-g-PCL(MSA):CG) or side chains terminated with sulfone groups (CS-SB-SFD-g-PCL:CG).

### 3.3. Kinetics of Vitamin Release and Trans-Epidermal Absorption

**Figure 7:**
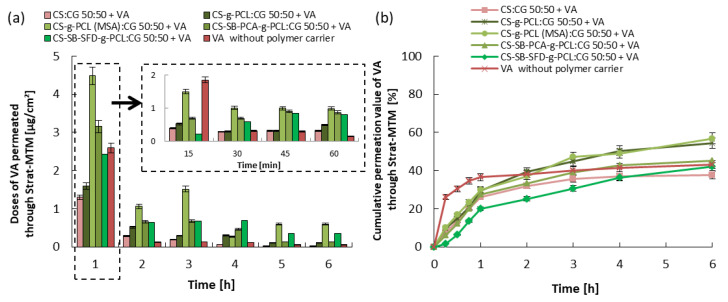
VA permeated through the Strat-MTM membrane: (**a**) 1 h doses and (**b**) cumulative release. Error bars represent the standard deviation of three replicates.

**Figure 8:**
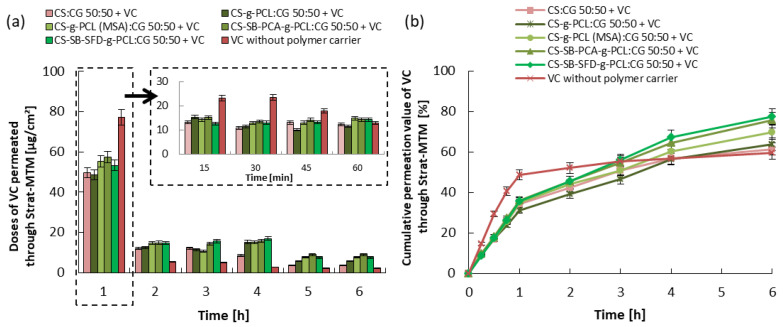
VC permeated through the Strat-MTM membrane: (**a**) 1 h doses and (**b**) cumulative release. Error bars represent the the standard deviation of three replicates.

**Figure 9:**
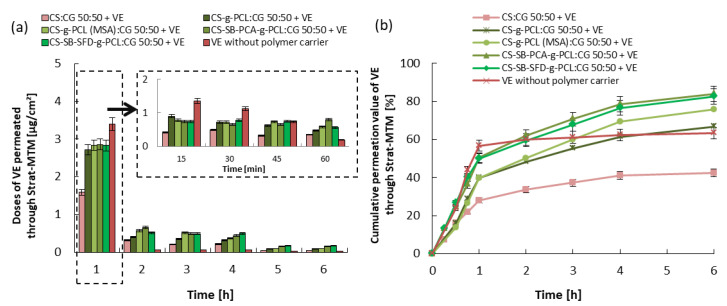
VE permeated through the Strat-MTM membrane: (**a**) 1 h doses and (**b**) cumulative release. Error bars represent the standard deviation of three replicates.

The release and skin permeation assays are important to evaluate the bioavailability and stability of the vitamin. Every test has been repeated three times, and the results are expressed by the mean value of the triplicates. In vitro conditions, the release of the vitamins from the cream containing the microcarrier without the microspheres was tested for 6 h, and the obtained results are shown in Figure 7, Figure 8 and Figure 9. In the case of vitamin release from the cream without a polymer carrier, initially, due to the burst effect, immediate release was observed, lasting 30 min for VA and 60 min for VC and VE, followed by an almost complete inhibition of release. The total % release of non-immobilized vitamins is 43% for VA, 60% for VC, and 65% for VE. Differences in the effectiveness of the release of these vitamins may result from various solubilities of the vitamins in water, their stability, and their ability to penetrate the skin (epidermis).

Considering the release of vitamins from the analyzed creams with microspheres formed from hydrogel blends (containing modified chitosan), a continuous release process is observed, without the sharp flattening of the curve observed above. The vitamin doses, measured every 15 min (Figure 7, Figure 8 and Figure 9), were very similar to each other for the first 1 h of the release process, and during the following hours were gradually decreasing, but for up to 6 h, they were still therapeutically significant [52,53,54]. Therefore, the expected distributed dosage of these substances through the membrane imitating the epidermis was achieved over a longer period. The total amount of vitamin released and permeated through the membrane during the total test time ranged from 40 to 55% for VA, 60 to 80% for VC, and 55 to 85% for VE, which significantly increased amount of the released and permeated vitamins by 15% and 20% compared to this same cream without loaded microspheres. The release of vitamins from a microcarrier made of a blend containing unmodified chitosan, i.e., from a material with a much lower SR, was much slower and was also in the first hour of this process. For this reason, the total amount of released and penetrated vitamins from obtained microspheres was the lowest despite the fact that this type of microspheres demonstrated the fastest weight loss over time.

**Figure 10:**
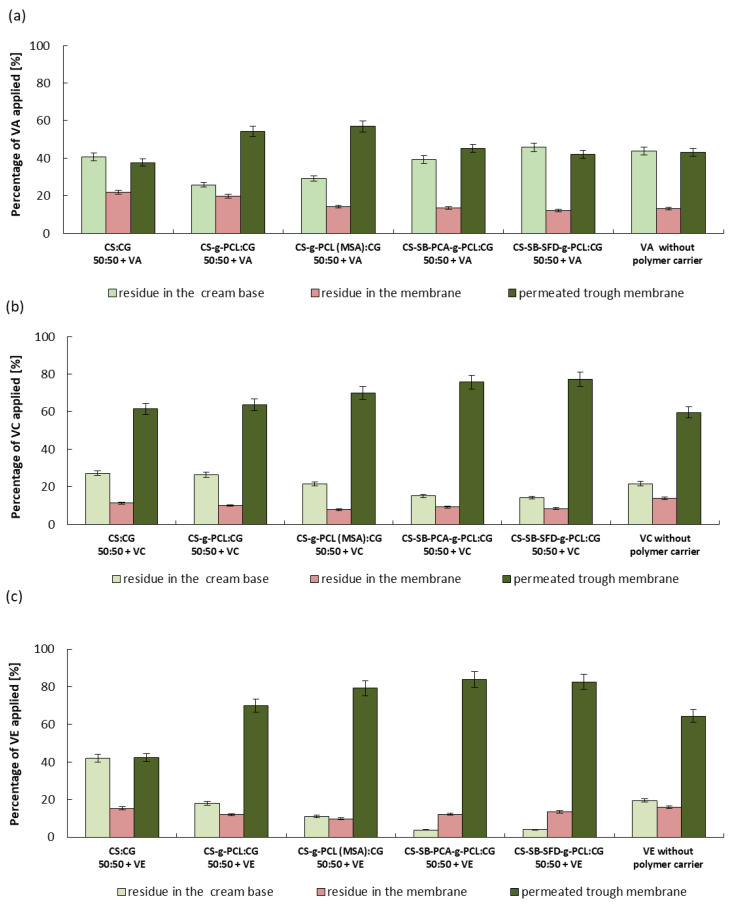
Relative vitamin content in the cream, membrane, and permeate after 6 h of the release process of VA (**a**), VC (**b**) and VE (**c**).

The observed difference in the total amount of vitamins released and penetrated is most likely related to differences in the process of penetration through the membrane of vitamins dispersed in the cream and released from the polymer microcarrier. Figure 10 demonstrates the amount of vitamins that remained in the cream and in the membrane and the amount of penetrated substance through the membrane. The largest amount of vitamins remained in the cream when microspheres were formed from a CS:CG 50:50 blend with the lowest swelling ratio and when a cream without a polymeric vitamin carrier was used. In the first case, the reason is the relatively slow rate of release of vitamins from the polymer matrix of the microspheres. In the case of the release of vitamins from a cream without the polymeric vitamin carrier, this phenomenon is more difficult to explain. Hydrogel microspheres are probably better transdermal permeation enhancers for the tested vitamins than the O/W emulsion present in the cream.

The in vitro release behavior indicates that the vitamins are loaded in the solid microparticle matrix rather than just distributed over the surface of the particles, which typically leads to an initial burst effect. This controlled release profile is important because it allows a specific amount of the active ingredient to be incorporated into the vehicle to be released over an extended period, reducing the need for repeated administration. Considering the use of these microcarriers in cosmetology and dermatology, the particles could provide an appropriate release profile of a specific vitamin after the application of the cream to the skin.

### 3.4. Assessment of Cytotoxicity

**Figure 11:**
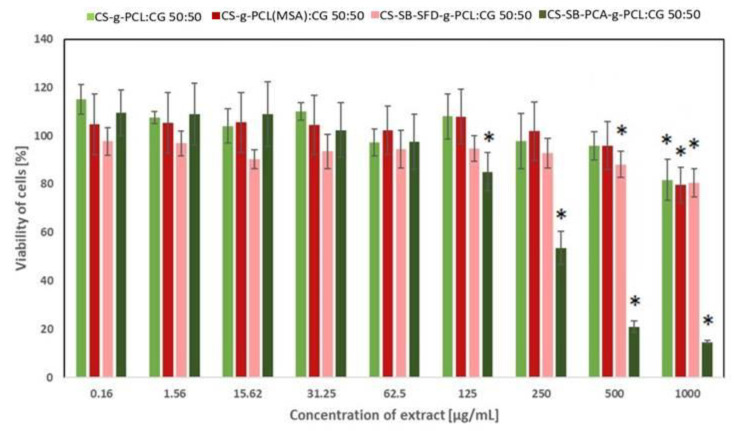
The effect of the developed blends on the viability of human fibroblasts (the results are shown as the mean ± SD; * *p* < 0.05 compared with the control).

**Figure 12:**
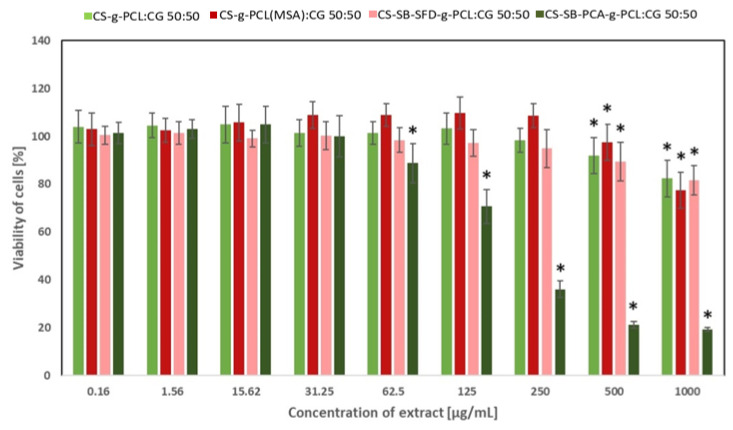
The effect of the developed blends on the viability of human keratinocytes (the results are shown as the mean ± SD; * *p* < 0.05 compared with the control).

The in vitro cytotoxicity of the developed blends for cosmetics applications has been analyzed against two cell lines: human fibroblasts and human keratinocytes with the use of the CCK-8 assay. The CCK-8 assay is a sensitive colorimetric technique used for the determination of the cell viability using WST-8 (2-(2-methoxy-4-nitrophenyl)-3-(4-nitrophenyl)-5-(2,4-disulfophenyl)-2H tetrazolium, monosodium salt) that is reduced by cellular dehydrogenases to an orange formazan product. The amount of the produced formazan is directly proportional to the number of living cells. The effect of the extract obtained from CS-g-PCL:CG 50:50, CS-g-PCL(MSA):CG 50:50, CS-SB-SFD-g-PCL:CG 50:50, and CS-SB-PCA-g-PCL:CG 50:50 on the viability of fibroblasts and keratinocytes is presented in Figure 11 and Figure 12, respectively. As shown in Figure 11, CS-g-PCL:CG 50:50 and CS-g-PCL(MSA):CG 50:50 did not affect the proliferation of cells at the concentration range of 0.16–500 µg/mL and C-SB-SFD-g-PCL:CG 50:50 at 0.16–150 µg/mL. This effect was expected when taking into account that the materials used for the synthesis of the blends did not exhibit cytotoxic effects, e.g., CS and CG (Figure S4), CS-g-PCL and CS-g-PCL(MSA) (Figure S5), and CS-SB-SFD and CS-SB-SFD-g-PCL (Figure S6). Some of the compounds even caused a slight increase in cells’ growth. The decrease in cells’ viability was only observed in the case of CS-SB-PCA-g-PCL:CG 50:50 at a concentration above 62.5 µg/mL (Figure 11) in the case of fibroblasts and above 31.25 µg/mL (Figure 12) in the case of keratinocytes. However, in this case, the compounds used for making the CS-SB-PCA-g-PCL:CG 50:50 blend, e.g., CS-SB-PCA and CS-SB-PCA-g-PCL, also caused the inhibition of the cell growth at a concentration above 62.5 µg/mL (Figure S7a) in the case of fibroblasts and at a concentration above 15.62 µg/mL (Figure S7b) in the case of keratinocytes. Thus, their addition to the blend also caused the cytotoxic effect of the final material at the highest concentration. The decreased cell viability at higher concentrations may be caused by the hydrolysis of the Schiff base and form a higher concentration of cytotoxic aldehydes because the formation of this imine is reversible in an aqueous solution.

## 4. Discussion

The obtained results prove that microparticles made of synthesized blends with immobilized vitamins A, C, or E can be successfully used as an additive not only in cosmetic creams but probably also in other preparations used in cosmetology or dermatology, applied externally to the skin. Taking into account the antibacterial properties and the release profile of all tested vitamins, microspheres obtained using CS-SB-PCA-g-PCL:CG 50:50 and CS-SB-SFD-g-PCL:CG 50:50 seem particularly promising for this type of application. The most toxic effect of these blends was found against *E. coli* and *S. epidermis*. Additionally, a blend containing modified chitosan-containing sulfone groups (CS-SB-SFD-g-PCL:CG 50:50) was also strongly active against *S. aureus* [47]. Analyzing the presented vitamin release profiles, it was found that microspheres made of these blends provide the transdermal effect of these substances for a period of at least 6 h. Since these microspheres presented relatively low solubility in the medium, the release and penetration of vitamins through the endoderm model (weight loss below 20%) were caused by the high swelling ratio (SR in the range of 1300–1500%), which allows for the leaching of vitamins into the medium. This mechanism determines the release rate of the tested vitamins, which is strongly dependent on the vitamins’ solubility in water, especially those poorly soluble in water.

Vitamin delivery systems using the described carriers are more effective than traditional creams or emulsions because it turns out that hydrogel vitamin carriers are a better enhancer of vitamin release and penetration through the endoderm than the emulsion present in the applied cream containing these vitamins in free form. The use of CS-SB-PCA-g-PCL:CG 50:50 microspheres may raise some concerns due to the results of cytotoxicity tests.

Cytotoxicity testing is a widespread technique for evaluating the toxicity of cosmetic ingredients and products, which can be used in preclinical studies. Therefore, analysis of the cytotoxicity of the ingredients is a key parameter to be considered before including them for efficacy-based validations. Additionally, the in vitro assays for the safety evaluation of cosmetic ingredients and products respect the 3R’s principles of replacing animal use [55]. Cell-based testing systems have a few advantages over other methods for the validation of efficacy because they are cost-effective, adaptable for high throughputs, and yield results considerably faster than conventional in vivo methods [56]. Multiple mechanisms and cell types are involved in the induction of skin toxicological responses, e.g., skin irritation involves resident epidermal cells, dermal fibroblasts, and endothelial cells, as well as invading leukocytes that interact with each other under the control of a network of cytokines and lipid mediators [57]. Therefore, two kinds of cell types have been selected for this study, human fibroblasts and keratinocytes. The keratinocytes (HaCaT), an immortalized cell line, were used as a humaFn skin cell model for cosmetic applications [58]. The in vitro analysis demonstrated that the viability of cells exhibited in the presence CS-g-PCL:CG 50:50 and CS-g-PCL(MSA):CG 50:50 was not affected in the presence of at the concentration of 0.16–5100 µg/mL (fibroblasts) or 0.16–250 µg/mL (keratinocytes). The proliferation of both kinds of cells was not affected by CS-SB-SFD-g-PCL:CG 50:50 at 0.16–250 µg/mL. The most significant effect on the decrease in cells’ viability was observed for CS-SB-PCA-g-PCL:CG 50:50 because the initial compounds also caused some cytotoxicity. However, even this blend (CS-SB-SFD-g-PCL:CG 50:50) is not cytotoxic to fibroblasts at a concentration 0.16–62.5 µg/mL or keratinocytes at a concentration 0.16–31.25 µg/mL. Thus, the results highlight the possible use of the developed blends in cosmetic or pharmaceutical formulations.

## 5. Conclusions

This work aimed to develop innovative microparticles composed of two natural polymers of marine origin, namely, chitosan, and carrageenan, and to evaluate their potential use for the controlled release of immobilized vitamins from a basic cream base as anti-aging preparations. The developed microcarriers had sizes in the range of 10–20 µm, with a regular shape and a smooth surface. Using vitamins A, C, and E as active ingredients, the obtained microparticles showed a loading capacity ranging from 70 to 97% and an excellent ability to provide controlled release within 6 h with a tendency to continue releasing. Moreover, in vitro biological tests conducted using fibroblasts and keratinocytes proved that there was no cytotoxic impact of microparticles, which is crucial from the carriers’ biocompatibility point of view. The controlled release profile and lack of toxicity make these formulations strong candidates for many applications in biomedicine, pharmaceuticals, cosmetics, biotechnology, agriculture, and biosensing.

## Figures and Tables

**Table 1:** The blends and their abbreviations used in this work.

Blends	
CS:CG 50:50	chitosan-*blend*-(ĸ-carrageenan), 50:50 (*w*/*w*)
CS-g-PCL:CG 50:50	[chitosan-*graft*-poly(ε-caprolactone)]-*blend*-(ĸ-carrageenan), 50:50 (*w*/*w*)
CS-g-PCL(MSA):CG 50:50	[chitosan-*graft*-poly(ε-caprolactone) obtained with the presence of methanesulfonic acid]-*blend*-(ĸ-carrageenan), 50:50 (*w*/*w*)
CS-SB-PCA-g-PCL:CG 50:50	[chitosan-2-pyridinecarboxaldehyde-*graft*-poly(ε-caprolactone)]-*blend*-(ĸ-carrageenan), 50:50 (*w*/*w*)
CS-SB-SFD-gPCL: CG 50:50	[chitosan-sodium-4-formylbenzene-1,3-disulfonate-*graft*-poly(ε-caprolactone)]-*blend*-(ĸ-carrageenan), 50:50 (*w*/*w*)

**Table 2:** The loading efficiency of the vitamins.

Sample	Vitamins	LE [%]
CS:CG 50:50	A	96.5
	C	70.6
	E	96.7
CS-g-PCL:CG 50:50	A	93.6
	C	71.5
	E	91.0
CS-g-PCL(MSA):CG 50:50	A	93.4
	C	76.3
	E	92.2
CS-SB-PCA-g-PCL:CG 50:50	A	96.9
	C	72.6
	E	96.6
CS-SB-SFD-g-PCL:CG 50:50	A	97.4
	C	74.8
	E	97.1

## Data Availability

The data presented in this study are available upon request from the corresponding author.

## Supplementary Materials

The following supporting information can be downloaded at: https://www.mdpi.com/article/10.3390/polym16131815/s1, Figure S1. Standard curves of vitamins A, C, and E; Figure S2. The HPLC chromatograms of the standards of vitamins A, C, and E; Figure S3. The HPLC chromatograms of the tested cream sample with 10% of microspheres CS-SB-PCA-g-PCL:CG 50:50 + VA; Figure S4. The effect of the CS and CG on the viability of human fibroblasts (A) and keratinocytes (B) (the results are shown as mean ± SD; * *p* < 0.05 compared with control); Figure S5. The effect of the CS-g-PCL and CS-g -PCL(MSA) on the viability of human fibroblasts (A) and keratinocytes (B) (the results are shown as mean ± SD); Figure S6. The effect of the dCsSB-SFD and CS-SB-SFD-g-PCL on the viability of human fibroblasts (A) and keratinocytes (B) (the results are shown as mean ± SD; * *p* < 0.05 compared with control); Figure S7. The effect of the dCsSB-PCA and CS-SB-PCA-g-PCL on the viability of human fibroblasts (A) and keratinocytes (B) (the results are shown as mean ± SD; * *p* < 0.05 compared with control).
