# Supplementary material for: Microspheres Based on Blends of Chitosan Derivatives with Carrageenan as Vitamin Carriers in Cosmeceuticals
Source: Polymers (Basel). 2024 Jun 26;16(13):1815. doi: 10.3390/polym16131815 (PMC11244320; doi:10.3390/polym16131815)
Supplement: Supplementary file 1 [file polymers-16-01815-s001.zip › polymers-3057755-supplementary.pdf]

polymers

Supplements

Microspheres Based on Blends of Chitosan Derivatives with Carrageenan as Vitamin Carriers in Cosmeceuticals

Kamila Lewicka [1], Anna Smola-Dmochowska [2], Piotr Dobrzyński [1,2], Natalia Śmigiel-Gac [2], Katarzyna Jelonek [2], Monika Musiał-Kulik[2], Piotr Rychter [1*]

1 Faculty of Science and Technology, Jan Dlugosz University in Czestochowa, 13/15 Armii Krajowej Av., 6 42-200 Czestochowa, Poland; k.lewicka@ujd.edu.pl (K.L.); p.dobrzynski@ujd.edu.pl (P.D.); p.rychter@ujd.edu.pl (P.R.)
2 Centre of Polymer and Carbon Materials, Polish Academy of Sciences, 41-819 Zabrze, Poland; asmola@cmpw-pan.pl (A.S.-D.); ngac@cmpw-pan.pl (N.Ś.-G.); kjelonek@cmpw-pan.pl (K.J.); mm sial@cmpw-pan.pl (M.M.-K.)

* Correspondence: p.rychter@ujd.edu.pl

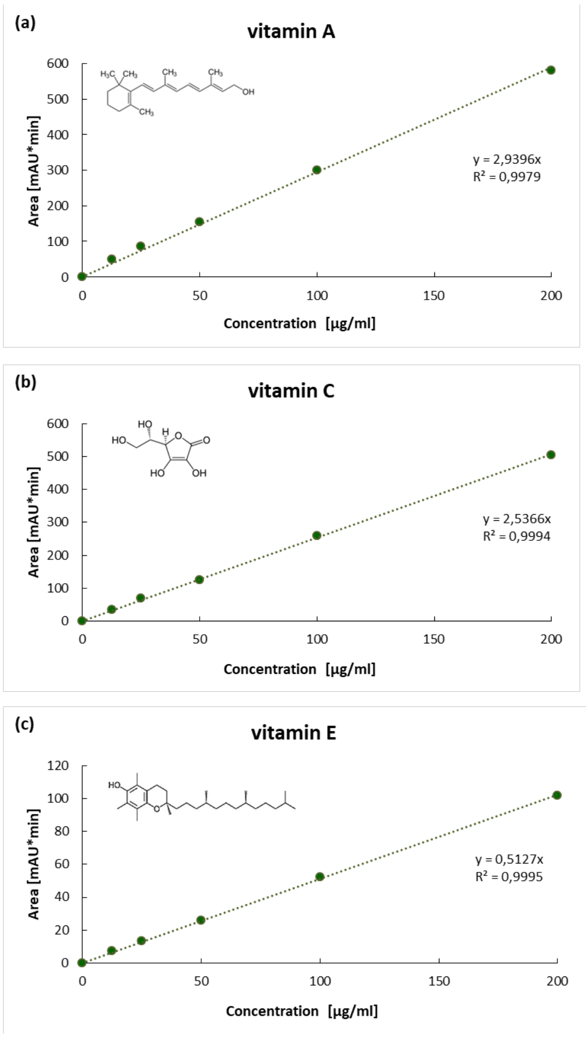

Figure S1. Standard curves of vitamins A, C, and E.

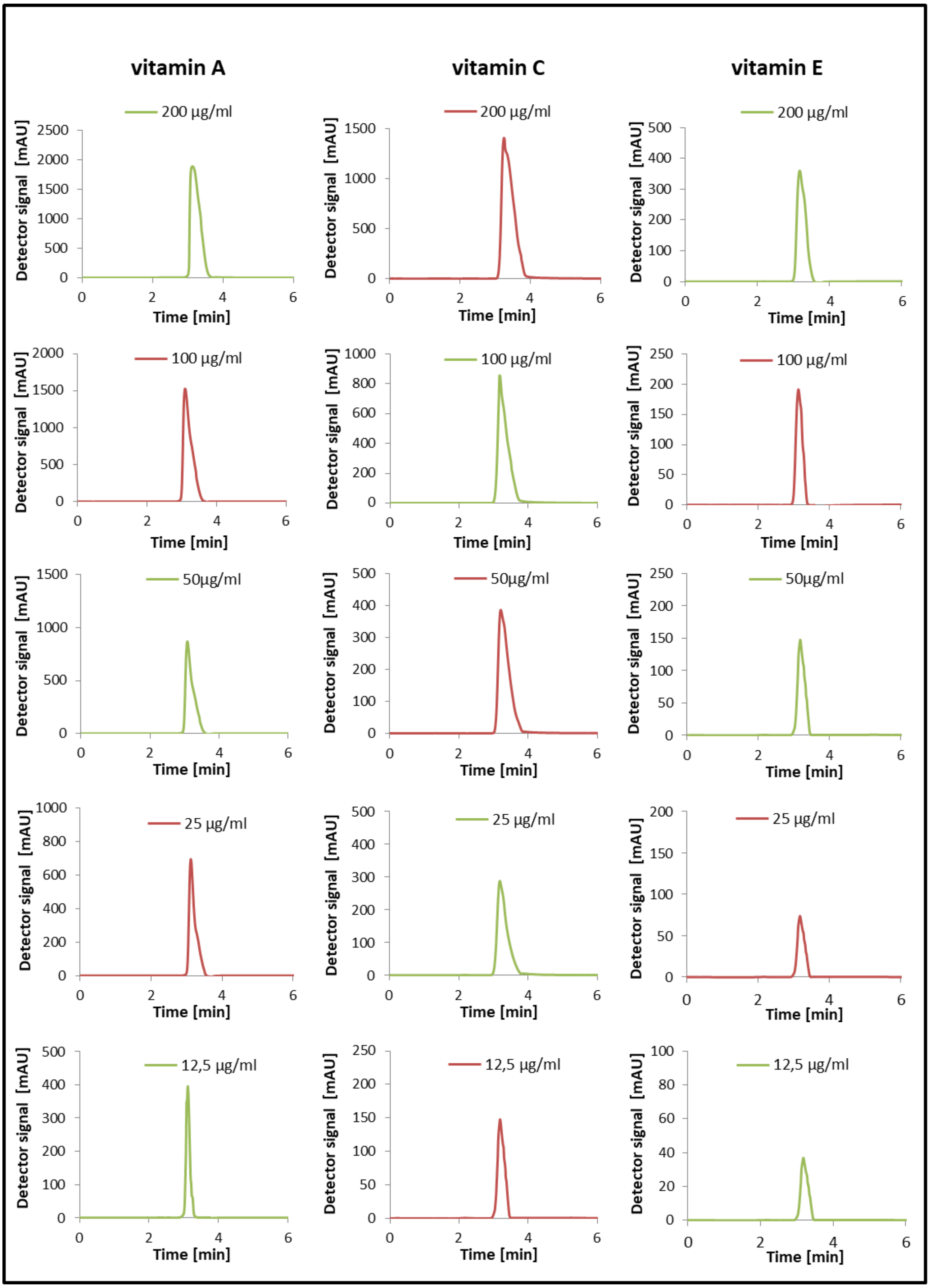

Figure S2. The HPLC chromatograms of the standards of vitamins A, C, and E.

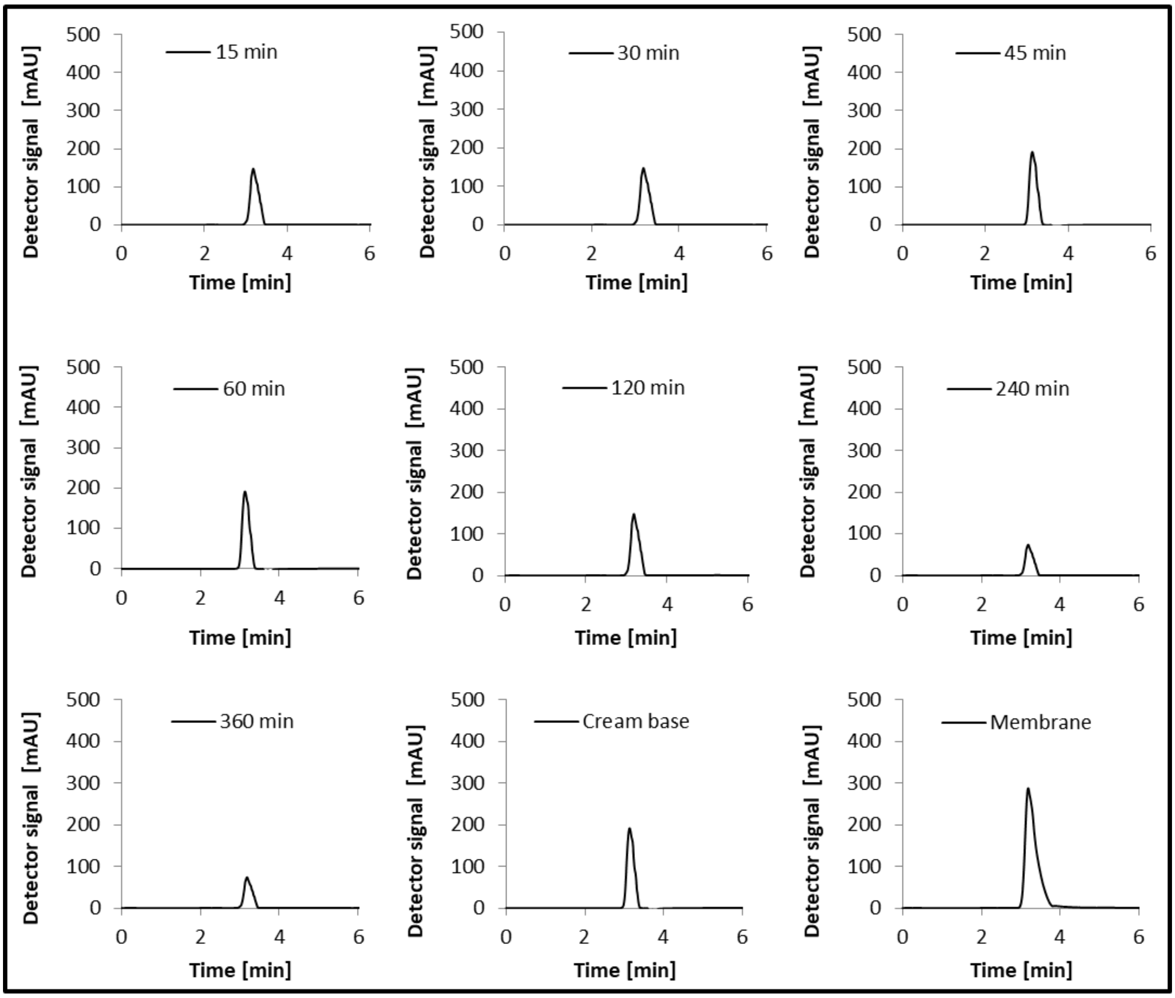

Figure S3. The HPLC chromatograms of the tested cream sample with 10 % of microspheres CS-SB-PCA-g-PCL:CG 50:50 + VA.

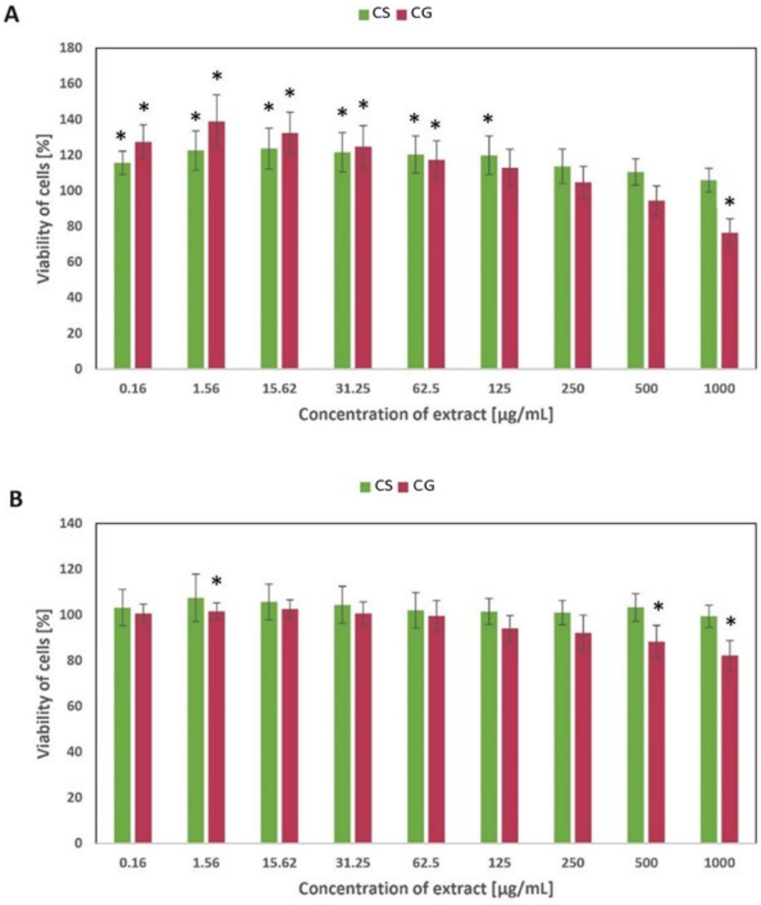

Figure S4. The effect of the CS and CG on the viability of human fibroblasts (A) and keratinocytes (B) (the results are shown as mean ± SD; *p < 0 .05 compared with control).

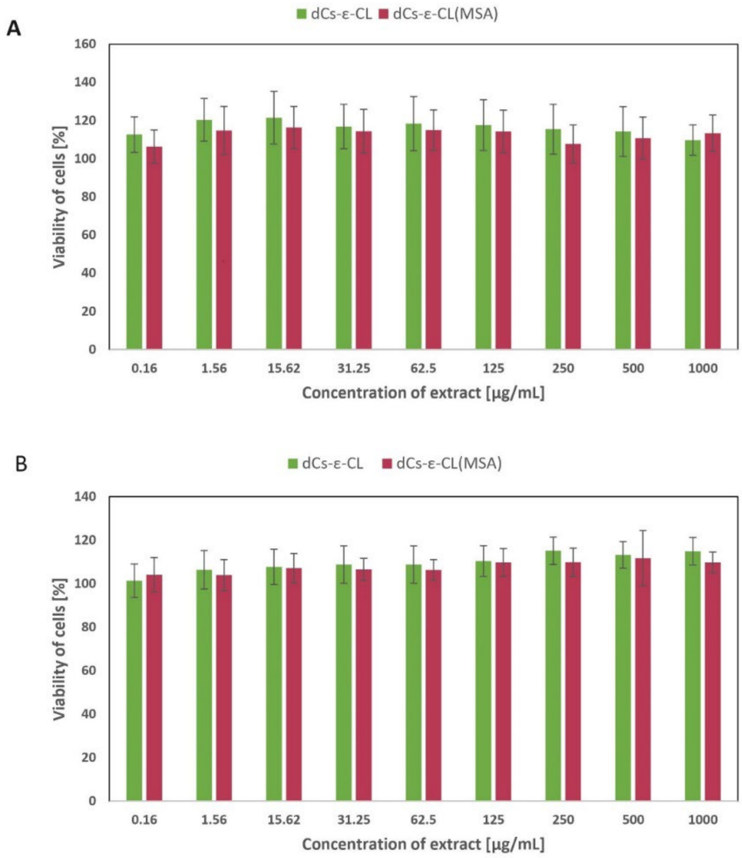
A
dCs-ε-CL
dCs-ε-CL(MSA)
Viability of cells [%]
Concentration of extract [µg/mL]
0.16
1.56
15.62
31.25
62.5
125
250
500
1000
B
dCs-ε-CL
dCs-ε-CL(MSA)
Viability of cells [%]
Concentration of extract [µg/mL]

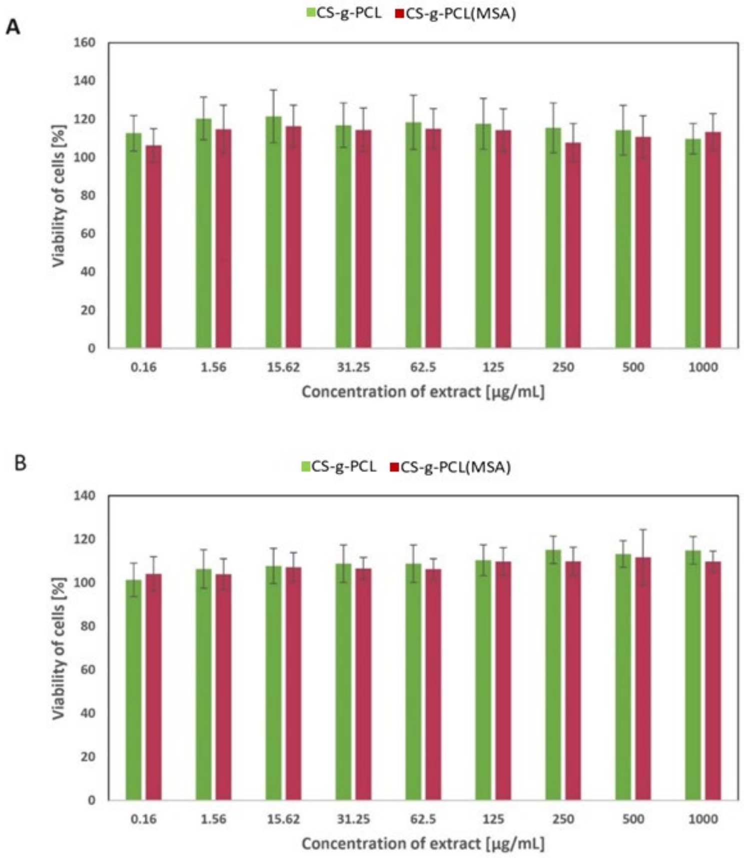

Figure S5. The effect of the CS-g-PCL and CS-g -PCL(MSA) on the viability of human fibroblasts (A) and keratinocytes (B) (the results are shown as mean ± SD).

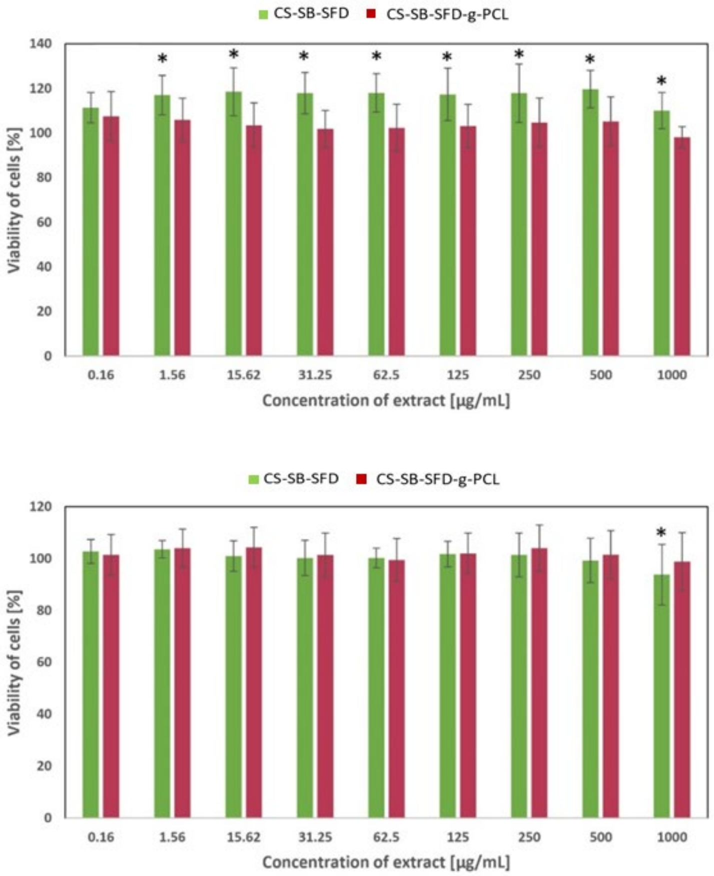

Figure S6. The effect of the dCsSB-SFD and CS-SB-SFD-g-PCL on the viability of human fibroblasts (A) and keratinocytes (B) (the results are shown as mean ± SD; *p < 0 .05 compared with control).

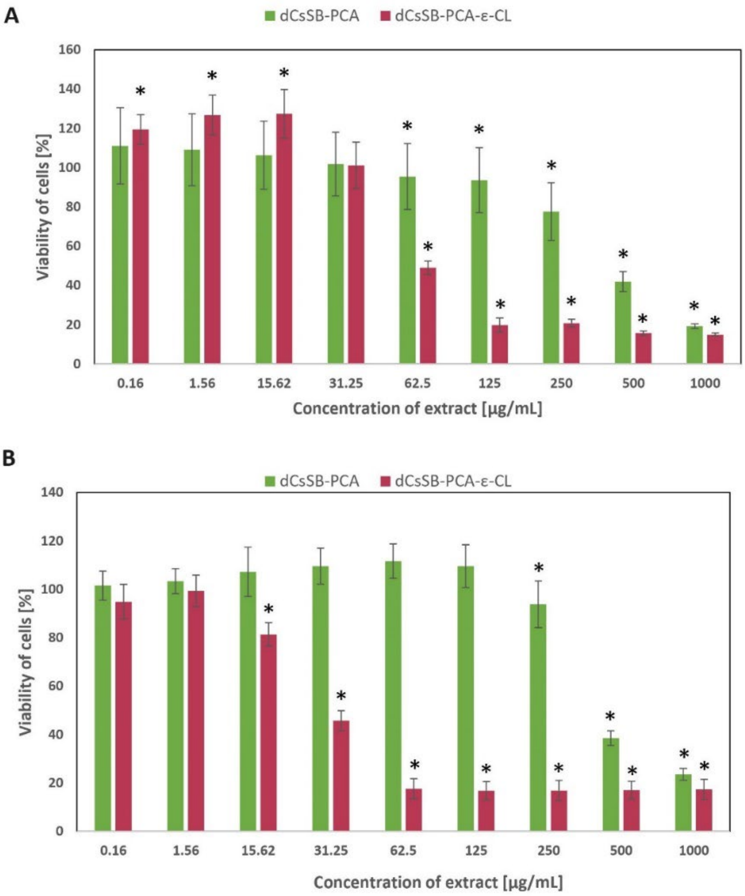
A
dCsSB-PCA
dCsSB-PCA-ε-CL
Viability of cells [%]
Concentration of extract [µg/mL]
0.16
1.56
15.62
31.25
62.5
125
250
500
1000
B
dCsSB-PCA
dCsSB-PCA-ε-CL
Viability of cells [%]
Concentration of extract [µg/mL]

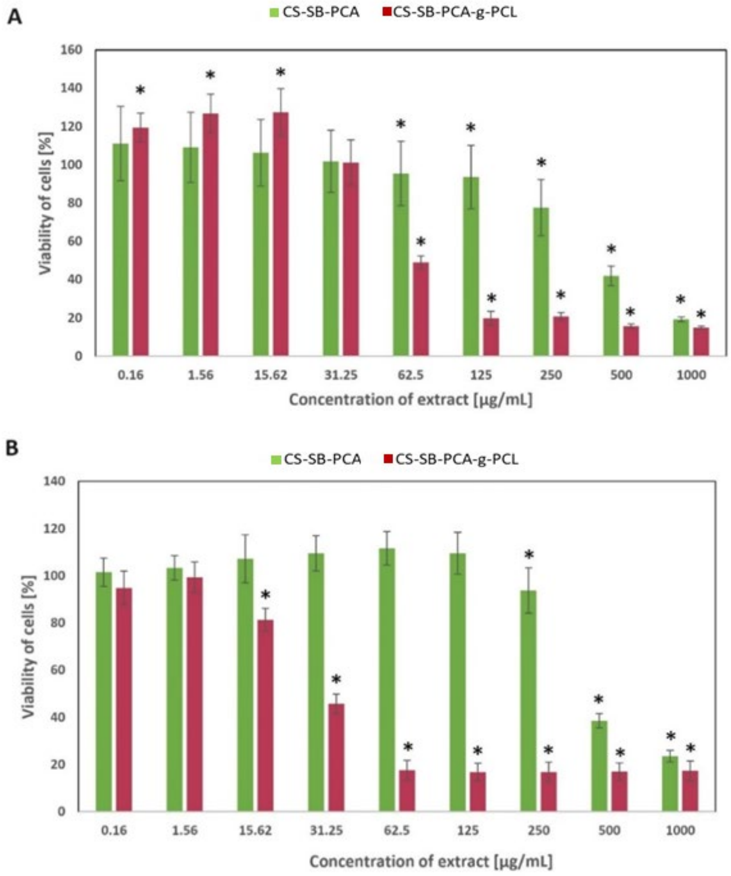

Figure S7. The effect of the dCsSB-PCA and CS-SB-PCA-g-PCL on the viability of human fibroblasts (A) and keratinocytes (B) (the results are shown as mean ± SD; *p < 0 .05 compared with control).

Disclaimer/Publisher's Note: The statements, opinions and data contained in all publications are solely those of the individual author(s) and contributor(s) and not of MDPI and/or the editor(s). MDPI and/or the editor(s) disclaim responsibility for any injury to people or property resulting from any ideas, methods, instructions or products referred to in the content.
